# Effect of Using Rotational and Static Kilns on the Properties of Eco-Friendly Lightweight Aggregates Made with Pumice Scraps and Spent Coffee Grounds

**DOI:** 10.3390/ma18153692

**Published:** 2025-08-06

**Authors:** Fabiana Altimari, Fernanda Andreola, Isabella Lancellotti, Carlos Javier Cobo-Ceacero, Teresa Cotes-Palomino, Carmen Martínez-García, Ana Belen López-García, Luisa Barbieri

**Affiliations:** 1 Department of Engineering “Enzo Ferrari”, University of Modena and Reggio Emilia, via Vivarelli, 10, 41125 Modena, Italy; fabiana.altimari@unimore.it (F.A.); fernandanora.andreola@unimore.it (F.A.); isabella.lancellotti@unimore.it (I.L.); 2 CRICT—Inter-Departmental Research and Innovation Center on Constructions and Environmental Services, 41125 Modena, Italy; 3 Department of Chemical, Environmental and Materials Engineering, Higher Polytechnic School of Linares, University of Jaén, Campus Científico-Tecnológico de Linares, 23700 Linares-Jaén, Spain; cjcobo@ujaen.es (C.J.C.-C.); mtcotes@ujaen.es (T.C.-P.); cmartin@ujaen.es (C.M.-G.); ablopez@ujaen.es (A.B.L.-G.)

**Keywords:** sintering methods, lightweight aggregates (LWAs), porous ceramic materials, spent coffee grounds, pumice rock, red clay

## Abstract

In this work, lightweight aggregates (LWAs) were prepared from an Italian red clay, pumice scraps, and spent coffee grounds. Chemical and physical characterization was first performed on the raw materials and then on the finished products. By studying the thermal behavior of the materials, the correct firing temperature was evaluated. The obtained aggregates were fired in two different modes: in a rotary kiln and in a static kiln; the influence of the firing processes on the finished products was assessed. This study can be useful for industrially scaling up this process. Firing in a rotary kiln reduced the average diameter of the aggregates (negative expansion index), resulting in a higher compressive strength and dry particle density compared to an aggregate containing only clay. The pH and electrical conductivity values address their use in agronomy without causing problems to crops, while the higher compressive strength, density, and porosity values could allow their use in construction.

## 1. Introduction

The steady growth in world population with the consequent increase in anthropogenic activity has led to the overexploitation of the natural resources on which the quality of life is based: water, soil, and air. Intensive agriculture leads to serious consequences such as deforestation, desertification, groundwater pollution due to the massive use of pesticides and fertilizers, air pollution due to high ammonia emissions, etc. [1]. The environmental and social consequences of land degradation such as desertification, especially in the Mediterranean regions, have been known and analyzed for some time now [2,3,4]. Soil erosion is more pronounced during periods of heavy rainfall as topsoil is carried elsewhere and there is a loss of nutrients and reduced infiltration. There is a need to increase or maintain vegetation cover in these areas to mitigate the problem [5].

Rethinking the entire global agricultural system becomes mandatory; we need to discard the idea of intensive agriculture and focus on a system to make soils fertile and increase infiltration at the expense of surface runoff.

Based on these assumptions, many studies have been conducted in recent years on the production of porous ceramic materials such as artificial lightweight aggregates (hereafter referred to only as lightweight aggregates or LWAs). Due to their chemical and physical properties, lightweight aggregates are suitable for numerous applications, such as energy conversion and storage [6], heat transfer [7], green roofs, rooftop gardens, urban greening, and hydroponics [8]. The technical reference definition for lightweight aggregates (LWAs) is contained in the standard for concrete [9] and it defines lightweight aggregates as any “aggregate of mineral origin having a density of granular particles [10] less than 2000 kg/m^3^ or having a loose bulk density [11] less than 1200 kg/m^3^”. The main constituent is (almost) always clay: if it is subjected to high temperatures (commercial LWAs are manufactured by firing in rotary kilns at maximum temperatures of 1000 to 1300 °C, the temperature will vary depending on the specific material being processed and the desired reaction [12]), it tends to expand its starting volume a lot (even up to 6–7 times), releasing gas phase components (organic substances and reduced metal oxides), resulting in a honeycomb-like internal structure. According to a study conducted by the authors, the increase in volume is more related to the formation of an adequate viscous phase than to the release of gas [13].

In the agronomic field, the usefulness of LWAs is mainly related to the open porosity of their structure, which allows water to drain efficiently, preventing water stagnation and thus the possible proliferation of mold and/or fungal diseases; it can also be useful for reducing the weight of the substrate (by about 10%), in the case of green roof use, and for mulching [14].

This work stems from the idea of obtaining a green alternative to current commercially available LWAs for agricultural purposes. This alternative would use less virgin clay, partially replacing it with reclaimed materials. The effect of static or rotary firing on the physical and chemical properties of the prototypes will also be compared. The raw materials used are Italian red clay, pumice, and spent coffee grounds. The authors have previously used pumice stone to manufacture LWAs through powder sintering in a static electric furnace [15]. Pumice scraps were selected to enhance the light weight and the porous structure compared to red clay LWAs. It should be noted that developing lightweight aggregates for agronomic applications presents several challenges. Controlling the porosity and water absorption is essential to ensure a proper balance between drainage and water retention, thus avoiding stagnation, which is harmful to roots. Maintaining a neutral–sub-acidic pH (pH = 6.0–6.9) and low electrical conductivity is also necessary to avoid hindering the absorption of nutrients. The aggregates must be light yet resistant and stable over time, as well as being compatible with different cultivation systems, including hydroponics. Although the use of waste materials is sustainable, it involves variability in quality and behavior during sintering, which must be optimized to ensure consistent performance and low environmental impact. Compliance with agronomic regulations is essential for the safe and legal use of the product.

Clay and volcanic ash have always been used for the production of LWAs due to the characteristics of the material obtained after firing. There are studies in the literature evaluating the geotechnical properties of clay LWAs [16,17,18], the mechanisms of expansion during firing [13,19], the effects of adding sand to smectite-rich Tunisian clay rocks [20], the use of zeolitic tuffs [21], and the use of volcanic pumice and lapillus [15]. There are also studies that used wastes of various kinds such as spent coffee grounds [8,22], fly ash [23,24,25], sewage sludge [26], rice husk ash [27], cattle bone flour ash [28], and waste from municipal and industrial origins [29]. Further examples include the use of diatomite waste [30], red ceramic waste [31], industrial residues rich in aluminosilicates and carbonates [32], paraffin/titanium-bearing blast furnace slag phase change aggregate [33], PET plastic waste and byproduct additives [34], as well as slag and fly ash for the stabilization of organic clay [35].

The literature is rich in insights into the design of lightweight aggregates using waste, and this has enabled the design of new environmentally friendly materials with a matrix consisting of clay and pumice and with spent coffee ground as the pore-forming agent. Thanks to the raw materials used, the aggregates could be defined as eco-friendly. The advantages of using pumice include its consistent chemical and mineralogical composition over time and the fact that it is a national byproduct with limited marketing potential that risks becoming waste. The benefit of using spent coffee grounds is that they are a waste that is easily available in any geographical area and burned during the cooking process, increasing the porosity of the material. Two different forms of firing for LWAs were evaluated, in a static kiln and in a rotational kiln, to highlight the advantages and disadvantages of the two sintering processes. The production of expanded clay involves a high-temperature firing process that transforms raw clay into a lightweight, porous material. The choice between a static and a rotary kiln for this process affects several aspects of production. In a static kiln, the clay LWAs are placed on fixed supports in a stationary firing chamber, and heat can be distributed by natural or forced convection; there can be temperature variability within the kiln, with some areas hotter than others. Thus, when firing in a static kiln, the expanded clay may have variations in porosity and density due to the uneven heat distribution. In this case, firing may take longer to achieve an optimum balance between densification degree and interconnected pores available for water retention. In a rotary kiln, the clay is placed in a drum that constantly rotates, ensuring that the material is continuously mixed and evenly exposed to heat. The final product tends to have a more uniform porosity and density, and firing is generally faster and more efficient. In general, rotary kilns are preferred for high volume production and to achieve uniform product quality, while static kilns can be used in smaller production environments or where uniform firing is not critical. Static firing has been considered, since there are many ceramic tile production plants in the national territory. In this context, if production is to be diversified or converted, the production of lightweight aggregates (LWAs) could be evaluated using equipment that is already available.

The scope of this work has been to characterize the new LWA formulations in terms of their physical, chemical, and mechanical properties in order to evaluate the best sintering process. The raw materials underwent characterization in terms of particle size distribution, moisture content, chemical composition (XRF and CHNS), mineralogical phases (XRD), and thermal behavior (TG/DTA for clay and pumice; calorimetry for SCGs). Formulations with varying clay contents (28–100 wt%) were prepared, mixed with water based on their consistency limits (liquid limit, plastic limit, and plasticity index), shaped into spherical pellets, and fired using both rotary and static kilns. The LWAs were then characterized by measuring their loss on ignition (LOI); bloating index (BI); bulk and particle densities; loose bulk density; total, open, and closed porosity; water absorption after 24 h (WA24h); compressive strength (CS); pH; and electrical conductivity (EC).

## 2. Materials and Methods

### 2.1. Raw Materials

The raw materials used during the experimentation were Italian red clay, which was extracted from the Samone quarry in the municipality of Zocca (Modena, Italy) and supplied by the company ESCAVAZIONI INDUSTRIALI BARONI s.r.l; pumice rock, which were processing and mining scraps less than 3–4 mm in diameter that were extracted from quarries located in Pitigliano and Tessennano around Bolsena Lake (Lazio, Italy), which were supplied by the company EUROPOMICE S.R.L; and spent coffee grounds of an 80% Arabica/20% Robusta blend from TORREFAZIONE MUSETTI of Pontenure (Piacenza, Italy), which were collected from a series of bars.

The raw materials were initially sieved or ground using a Restch^®^ SK 100/C Spezialstahl arm mill (Retsch GmbH, Haan, Germany), producing grains less than 200 μm in diameter, which were then dried in an oven at a temperature of 100 ± 5 °C for 24 h to remove any moisture. Characterization of these grains was carried out to assess their suitability for use as a matrix or pore-forming agent.

### 2.2. Characterization of Raw Materials

#### 2.2.1. Particle Size and Moisture Content

The particle size of the pumice rock and spent coffee grounds was measured using a laser diffraction particle size analyzer (Malvern Instruments, model Hydro200S). This analysis was not performed on the clay since it is sold already divided into particle size classes. The results were reported using the equivalent spherical diameter as a reference. The characteristic diameters D_10_, D_50_, and D_90_ correspond to the diameters below which 10%, 50%, and 90% of the distribution is found.

The grain size analysis showed that the pumice and spent coffee grounds (SCGs) had very different grain sizes. The pumice turned out to be finer than the SCGs, as can be seen in Table 1.

After evaluating the grain size, the raw materials were weighed, dried, and weighed again in order to evaluate the moisture content of the samples. The average moisture content was 4% for the clay, and 2.6% and 4.8% wt for the pumice and spent coffee grounds, respectively.

Moisture content (WC) was calculated according to standard UNE EN 103300:1993 [36] using three samples of about 30 g for each raw material.

The moisture values of the samples did not turn out to be very high, although clay is known to be absorbent. The highest average moisture value was found in the spent coffee grounds, but it was still an acceptable value for workability purposes.

#### 2.2.2. Chemical and Mineralogical Analysis

Assessing the presence of chemical elements of interest is critical when new formulations are to be studied. Elemental analysis made it possible to assess the nitrogen (N), carbon (C), hydrogen (H), and sulfur (S) contents, which were expressed as a percentage of the initial weight of the analyzed sample.

Elemental analysis was performed using an elemental analyzer (Leco Truspec Micro CHNS) coupled with an oxygen module. Chemical analyses were performed with an ARL PERFORM’X model sequential X-ray fluorescence (XRF) spectrometer. The Loss Of Ignition (LOI) was calculated as the weight difference before/after calcining the materials under study at 1000 °C for 2 h.

The chemical and elemental analysis results are shown in Table 2 and Table 3, respectively.

Chemical analyses of the clay and pumice showed that they are alumino-silicate materials with a high iron oxide content, which is mainly present in clay and is responsible for its typical red color. Pumice also had a good amount of potassium oxide. Due to their silica and alumina contents, they were chosen as matrix components for LWAs since they can stabilize ceramic bodies during firing. Chemical analysis of the spent coffee grounds confirmed that it is a purely organic material; this confirmed that it can be used as a pore-former at the firing temperature of LWAs.

Elemental analysis was performed on the clay and spent coffee grounds given the high LOI value. From the analysis conducted on the clay, the presence of carbon and hydrogen in amounts less than 1% was noted. From the analysis conducted on the coffee, a significant presence of carbon and oxygen was observed, in agreement with other authors. The oxygen comes from carbohydrates and lipids while the nitrogen comes from proteins [37].

The utilization of SCGs as a filler in the production of LWAs has been studied and has demonstrated promise in improving soil characteristics and introducing valuable nutrients, thereby enhancing plant growth [8,38]. The unique chemical composition of SCGs substantially contributes to the quality of the resulting lightweight aggregate. SCGs contain significant amounts of nitrogen, potassium, and other essential nutrients that benefit soil health, acting as a slow-release fertilizer [39]. The calcined SCGs also maintain their potassium, calcium, and macronutrient contents after firing. This feature can lead to improved growth rates and yields of nursery grapevines and other agricultural crops, facilitating a sustainable approach for soil management [38]. Furthermore, the porosity introduced by SCGs into the aggregates can enhance water retention and aeration in soils, which are critical factors for plant health and growth [8]. Research conducted by Andreola et al. showed that SCGs can partially replace traditional clay in lightweight clay ceramic aggregates, where they acted as a pore-forming agent before firing [8]. This method not only reduces the overall environmental impact associated with producing traditional aggregates, but also introduces an effective buffering capacity in soils, mitigating leaching and nutrient loss [38]. The Life Cycle Assessment (LCA) approach highlights that using waste materials such as SCGs can significantly lower the carbon footprint of construction materials while maximizing resource utilization [40].

Mineralogical analyses were performed with an X-ray diffractometer (X’PERT 3 POWDER PANALYTICAL model). Further analysis of the clay (ethylene glycol vapor test for 8 h at 60 °C and thermal treatment at 550 °C for 2 h) was carried out to better identify the clay mineral species.

**Figure 1:**
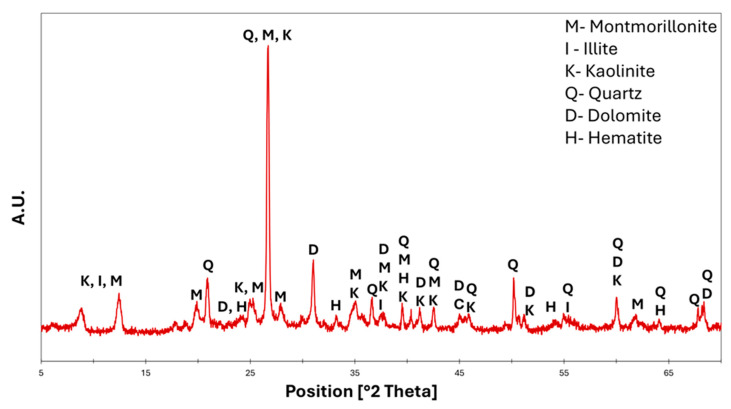
Mineralogical analysis of red clay.

Qualitative mineralogical analysis performed on the clay (Figure 1) showed a spectrum with defined peaks at lattice distances that can be related to the following crystalline phases: quartz (SiO_2_, ref. code 01-078-1252), kaolinite (Al_2_Si_2_O_5_(OH)_4_, ref. code 00-001-0527), illite (KAl_2_Si_3_AlO_10_(OH)_2_, ref. code 00-003-0010) clays, expandable smectite minerals (e.g., montmorillonite, (Na,Ca)_0.33_(Al,Mg)2Si_4_O_10_(OH)_2_·nH_2_O), ref. code 00-012-0219), calcium and magnesium carbonate (dolomite ((CaMg)CO_3_), ref. code 01-083-1766), and hematite (Fe_2_O_3_, ref. code 01-089-0598), confirming the presence of high amounts of iron. The plastic function of the clays enables the processing of the mixture and gives it mechanical strength. The carbonates, on the other hand, contribute to sintering and the formation of crystalline phases after firing, while quartz contributes to vitrification if it is in a fine fraction; if it is in a coarse fraction, it helps to control the shrinkage of the material during firing by counteracting any breakage.

The pumice used in this study was a mainly amorphous material (79%), making it suitable for firing. It consists of alkali feldspars rich in potassium, such as sanidine, confirming the high content of K_2_O in the chemical analysis. The mineralogical characteristics were determined and discussed by the same authors in a previous study [41].

**Figure 2:**
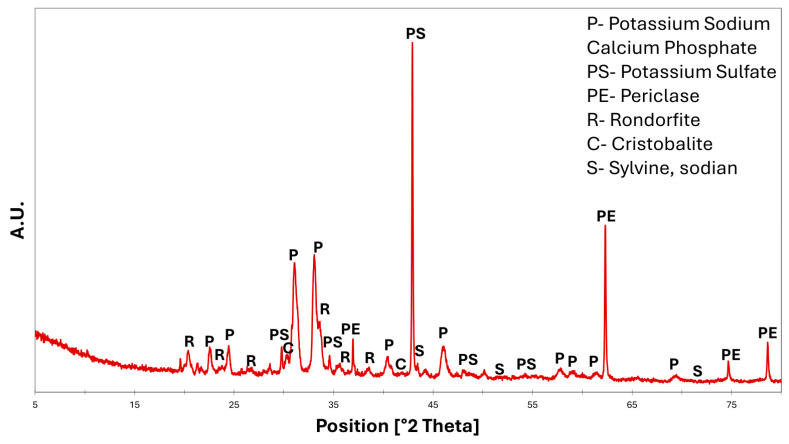
Mineralogical analysis of calcinated spent coffee grounds.

Mineralogical characterization of the calcinated spent coffee grounds (Figure 2) showed several diffraction peaks corresponding to the presence of potassium, sodium, and calcium phosphates (ref. codes: 00-051-0580 (potassium sodium calcium phosphate), 00-044-1414 (potassium sulfate), 01-075-0447 (periclase), 00-041-0248 (rondorfite), 01-089-3435 (cristobalite beta), 01-075-0300 (sylvine sodium)).

Properly defining formulations requires a chemical and mineralogical analysis of the raw materials. Understanding the results from these analyses enables the estimation of potential reactions during firing. Together with data from thermal analyses and the literature, this information can be used to identify the optimal process temperatures.

#### 2.2.3. Thermal Analysis

The materials will be subjected to heat treatment, so it was necessary to understand how they behave over time when subjected to a heating cycle. The thermal characterization techniques used were thermogravimetric analysis (TG) and differential thermal analysis (DTA) for the clay and pumice rock, and Mahler calorimetry for the spent coffee grounds.

TG and DTA were conducted with a NETZSCH STA 429 simultaneous thermal analyzer (CD) after grinding the samples into finer particles (average particle diameter of less than 20 μm); the analysis was performed in air at a heating rate of 10 °C/min.

Spent coffee grounds are organic materials, and they could release energy in the sintering phase by changing the energy requirements; therefore, calorimetric analysis is essential. The heating value was obtained according to the standard [42] by entering a series of data, obtained by subjecting three samples (about 1 g by weight) to Mahler’s calorimetric bomb test, into a numerical model.

**Figure 3:**
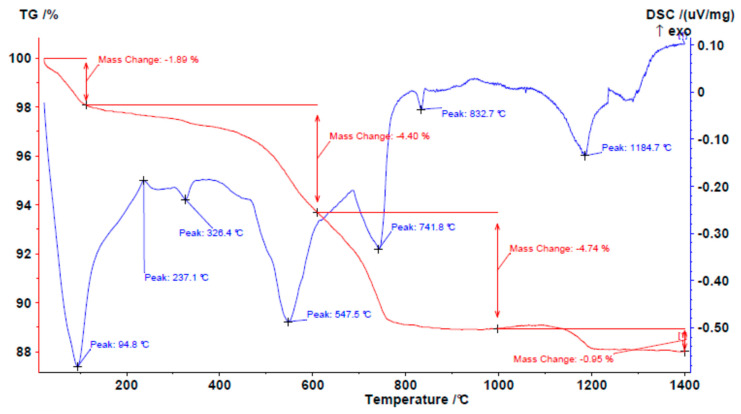
TG/DTA of clay.

The results obtained for the clay (Figure 3) show that according to the DTA (blue curve):

- At 100–200 °C, an endothermic phenomenon occurs with the loss of any absorbed water;
- Around 250 °C, an exothermic phenomenon related to the combustion of organic matter occurs;
- There is a peak around 328 °C; this endothermic phenomenon is probably related to the loss of interlayer water from the expanded clay minerals;
- At around 550 °C, dehydroxylation occurs, resulting in the loss of structural OH^−^ groups;
- At 700–900 °C, the decomposition of dolomite occurs in two stages;
- At 1184 °C, the melting of the crystal lattice occurs.

The TG (red curve) shows that at the aforementioned peaks, there was a change in mass (%) due to the loss of moisture (1.80%), decomposition of the organic matter (~0.50%), the loss of interlayer water (~0.50%), dehydroxylation (4.40%), and the decomposition of the dolomite (about 5%).

**Figure 4:**
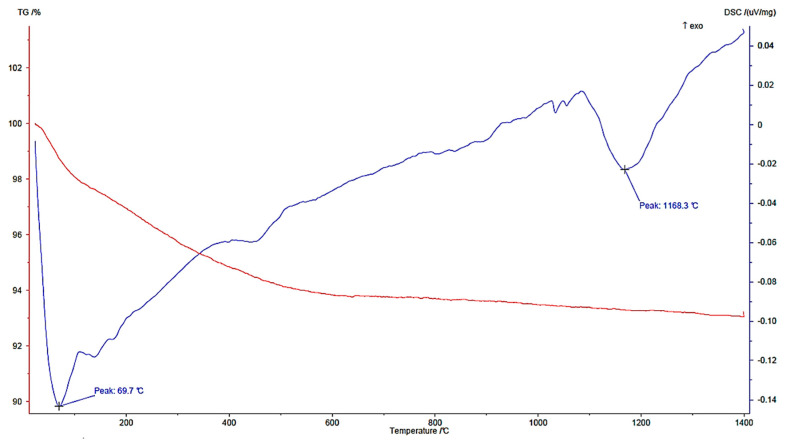
TG/DTA of pumice.

From the thermal analysis performed on the pumice (Figure 4), an endothermic event around 100 °C due to free water loss was seen; at this point, the pumice sample had already lost about 6% of its initial weight. This significant amount of initial moisture was probably due to the high moisture content in the extraction quarry. The next peak, around 1200 °C, was due to the melting of the crystal lattice.

The spent coffee grounds were subjected to Mahler’s calorimetric bomb test to predict their eventual firing behavior by evaluating their calorific value (Table 4). The reported values are similar to those reported in the literature [43,44].

### 2.3. Sample Preparation

The raw materials were characterized and then the compositions shown in Table 5 were used to produce LWAs, which were identified by the letter A (aggregates) and two numbers indicating the percentage of clay in the composition.

The amount of SCGs was chosen based on previous studies carried out by the authors [8].

The samples were prepared by drying the raw materials in an oven at a temperature of 100 ± 5 °C for 24 h and reducing their particle size to a diameter of less than 200 μm so that they could be easily mixed. Next, the raw materials were mixed, and water was added. The amount of water for each composition was a function of the consistency limits of the formulations themselves [45,46,47]. The resulting mixture was covered and left to stand for 24 h. After the required time had passed, the material was placed inside an air-powered extruder (Nannetti Pneumatic Extruder) to obtain cylinders with a diameter of 1 cm. The cylinders were divided into sections about 7 mm in length, and each individual section was processed manually, obtaining spheres. Efforts were made to avoid the formation of surface cracks by placing the aggregates in an oven for 24 h at 100 ± 5 °C to remove any moisture before firing.

The firing of the spheres took place in a rotary kiln (Nannetti^®^ TOR- R 120-14) that simulated, on a small scale, the firing methodology of expanded clay aggregates at the industrial level. Twenty-five samples were placed inside the kiln at a time and moved to spend the first 2 min in a preheating zone and the next 4 min in the hottest zone. After this period, the samples were removed from the back of the oven and allowed to cool.

The working temperature suitable for each composition was evaluated as a function of the firing temperature of a sample with only clay (A100) (1175 °C according to the literature) [47]. The optimum working temperature was the maximum allowed by the pellets; if the temperature is too high, these pellets adhere to each other and to the furnace tube. At this temperature, the maximum swelling that the granular material can undergo when it transforms into LWAs occurs. Given the presence of organic material, several attempts were made, decreasing the firing temperature by 5–10 °C from time to time until the optimal working temperature was reached; the optimal working temperature for each formulation is shown in Table 6 [48].

The dried specimens were also fired in a static furnace (Lenton Thermal Designs Ltd., Lenton FURNACES, Essex, UK); they were put directly into the furnace for one hour at a temperature of 1000 °C. This was performed to simulate the thermal shock that aggregates undergo during industrial processes (even though they use higher temperatures). The temperature depended on the thermal properties of the raw materials and was selected based on previous research by the authors [8]. The selection of the optimal sintering conditions must consider a suitable porosity, crush resistance (mechanical strength), and energy consumption. Changes in temperature did not lead to significant changes in the material properties. In addition, a further increase could have encouraged undesirable densification phenomena to occur. Once fired, the specimens were removed from the oven and allowed to cool in air through natural convection.

### 2.4. LWA Characterization

#### 2.4.1. Limits of Consistency

Consistency limits are normally determined for soils, but since clay is the main constituent of aggregates, they were evaluated to determine the correct amount of water to form pellets. The mixture was prepared by adding a known amount of water and once formed, the mixture was divided into two portions to make the liquid limit (LL) and plastic limit (PL) measurements. The difference between the LL and PL yields the plasticity index (IP), which precisely represents the moisture range within which the soil is in a plastic state. Depending on the plasticity index, the soil is classified as very soft (IP = 0–5), non-plastic (IP = 5–15), not very plastic (IP = 15–40), and very plastic (IP > 40) [49].

The liquid limit was calculated according to the standard; it was defined as the amount of water contained in a wet soil sample tested by Casagrande [50].

The plastic limit was calculated using two methods: according to the standard [51] and using the Bending Test.

The Bending Test is not a standard and was developed in a scientific study [45]. This method allows for the calculation of the plastic limit of a material as a function of two parameters: the “water content” (W) and the “bending at cracking” (B). Cylinders about 3 mm in diameter were formed and inserted into a profiler, forming exactly one cylinder with a length of 52 mm. The tool was moved back and forth until the cylinder cracked, and the distance between the flaps (D) was measured with a caliper. The W and B (Equation (1)) were used to calculate the WP.

(1) B=52.0−D

#### 2.4.2. Loss of Ignition

Sample weight loss (LOI) was evaluated to analyze the water content composition and organic matter content that was lost during heat treatment. This parameter was calculated using Equation (2), considering the initial weight (w_i_) and final weight (w_f_) of the sample (25 aggregates for each formulation) subjected to firing.

(2) $$LOI\left(\%\right)=\left(w_{i}-w_{f}\right)/w_{i}\cdot 100$$

#### 2.4.3. Bloating Index

Dimensional changes in the samples after heat treatment was calculated using Equation (3):

(3) $$BI\left(\%\right)=\frac{\left(d_{2}-d_{1}\right)}{d_{1}}\cdot 100$$

where d_1_ is the average sample size (25 aggregates for each formulation) before firing, d_2_ is the average sample size after firing, and BI is the bloating index.

The value of BI can also be indicative of the trend in changes in the total porosity.

#### 2.4.4. Density, Water Absorption, and Porosity

The bulk density (ρ_b_), dry particle density (ρ_Lrd_), and loose bulk density (ρ_app_) were calculated.

Bulk density (g/cm^3^) is the ratio between the mass of the sample and the volume it occupies including pores and the smallest cavities. Bulk density was calculated according to the standard [52].

Dry particle density (g/cm^3^) is the ratio of the sample mass to the volume occupied by the sample including closed pores and excluding pores accessible to water (open porosity).

Loose bulk density (LBD) (g/cm^3^) was calculated according to the standard [11]. Since the sample consisted of aggregates of different sizes, to account for the uncertainty, the measurements were repeated three times.

Water absorption (WA_24_) was calculated under static conditions after immersion of the sample (25 aggregates for each formulation) in water for 24 h according to the procedure described in [52].

In order to obtain as much information as possible for the characterization of the manufactured aggregates, open porosity, closed porosity, and total porosity were evaluated.

Total porosity (P_TOT_) was calculated as a function of the density of the matrix (true density, ρt) and dry particle density (ρLrd) using Equation (4):

(4) $$BP_{TOT}\left(\%\right)=\left[1-\frac{\rho_{Lrd}}{\rho_{t}}\right]\cdot 100$$

Open porosity (P_OPEN_) was calculated as a function of the bulk density and dry particle density using Equation (5):

(5) $$P_{OPEN}\left(\%\right)=\left[1-\frac{\rho_{Lrd}}{\rho_{bulk}}\right]\cdot 100$$

Closed porosity, i.e., the porosity of cavities not in communication with the outside, was calculated as the difference between total porosity and open porosity.

#### 2.4.5. Compressive Strength

The mechanical strength of the lightweight aggregates was determined using a one-axial compression test with a pneumatic press (Nannetti^®^ FM 96, Nannetti Spa, Faenza, Italy). This test measures the crush resistance (CS) of individual particles according to the method proposed by Yashima et al. [53]:

(6) $$CS=\frac{2.8\;PC}{\pi R^{2}}$$

PC is the fracture load, and R is the distance between the load points, which is the diameter of the LWA.

The result obtained refers to the maximum stress to which the aggregate can be subjected to before failure. To avoid the influence of the different sizes of the individual aggregates, the results obtained were averaged according to the height of the individual aggregate measured with a high-precision caliper. To obtain an average value, the test was performed on 25 spherical aggregates with a diameter of 9 ± 1.5 mm for each formulation.

This test is important to classify the aggregates according to possible uses.

#### 2.4.6. pH and Electrical Conductivity

The chemical properties of interest to assess the quality of the materials obtained were pH and specific electrical conductivity (E.C). Some of the possible fields of application of LWAs (e.g., green roofs and hydroponic crops) involve interaction with different types of plants; therefore, the most desirable pH and EC values must be lower than the threshold to avoid damage to the crops.

pH and electrical conductivity were measured in accordance with the standard rules [54,55].

Both tests were performed by placing 10 g of sample and distilled water inside a beaker, with a water-to-sample ratio of 2.5:1. The tests were performed under stirring (360 rpm) at room temperature for one hour. The solutions were filtered before the analysis.

The optimum pH and specific electrical conductivity values compatible with the possible agronomic uses of the aggregates were 6.5–7.5 for pH and 0.2–2 mS/cm for electrical conductivity [56,57,58]. The ranges of variation for the optimal pH and conductivity values depending on the type of culture can be seen in Table 7 and Table 8.

## 3. Results and Discussions

### 3.1. Characterization of LWAs

#### 3.1.1. Mineralogical Analysis

The first analysis performed on the aggregates was a mineralogical analysis to assess whether the different firing methods influenced phase formation. Two compositions were analyzed, one with the lowest clay content (28 wt%) and one with the highest (85 wt%). The compositions were also compared according to the firing processes (rotary kiln or static kiln).

**Figure 5:**
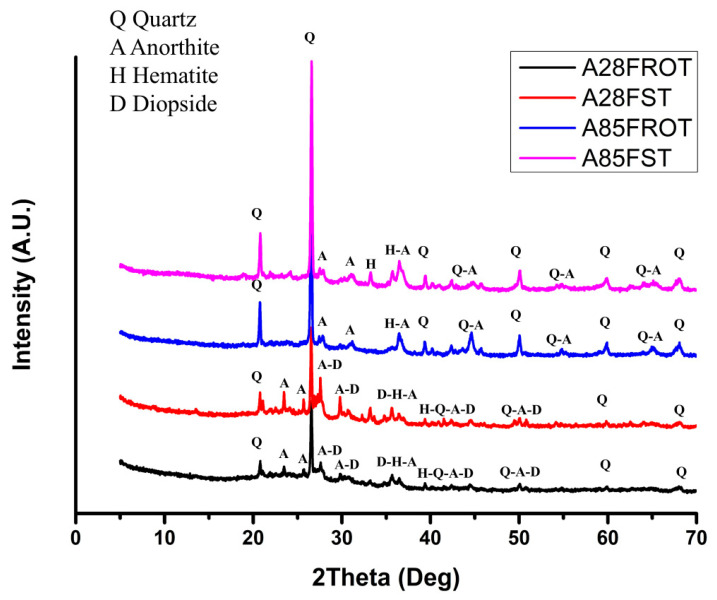
Comparative XRD of compositions containing 28% (A28) and 85% (A85) clay and fired using two different modes (ROT = rotatory kiln and ST = static kiln). Ref. codes: 01-083-0539 (quartz); 01-089-059 (hematite); 00-041-1486 (anorthite, ordered); 01-075-1092 (diopside).

As can be seen in Figure 5, the LWAs containing a higher amount of clay (A85) showed peaks with greater intensities, especially for quartz, indicating more robust crystalline formation under these conditions, which is directly correlated to the firing duration and temperature, in agreement with Abadel [61]. These variations suggest that the optimal conditions for producing lightweight aggregates can be tailored to harness specific material properties for enhanced performance in agriculture and construction applications.

Assessing the mineralogical effects of the presence of pumice instead of clay, it can be observed that in samples with 28% clay (static furnace), there were peaks of diopside and a band between 20° and 40°, which may be associated with the amorphous phase component present in pumice.

The difference between the two firing modes did not affect the crystalline phases that formed but rather the intensity of the peaks and some phases were well resolved with static kiln firing. This was probably due to the longer duration of the heating treatment compared to the rotational treatment.

#### 3.1.2. Consistency Limits and Optimal Amount of Water

Subsequently, the consistency limits of the compositions were evaluated before characterizing the aggregates; this was performed to assess the optimal amount of water to be added to the powders to obtain a mix with the right plasticity.

**Figure 6:**
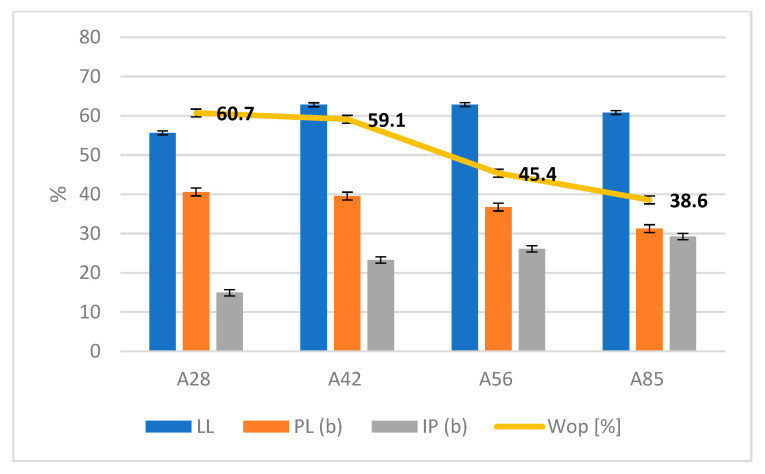
Consistency limits (liquid limit (LL), plastic limit (PL), plasticity index (IP), and optimal water quantity (Wop)).

In Figure 6, it is possible to see the changes in the liquid limit (LL), the plastic limit calculated using the Thread Bending Test method (PL (b)), the plasticity index (IP (b)), and the optimal amount of water (Wop) as the formulations varied. It can be seen that the workability of the mix increased as the clay content increased, and the water content required decreased at the same time. The variability of the data was not very relevant since it was less than 1%. The variability may also depend on the processing time, since the manual operation time may not be exactly the same for all the formulations, which could affect the water content.

#### 3.1.3. Weight Loss and Bloating Index

The samples were fired in two different kilns to evaluate the influence of the firing mode on the properties of the finished product. Table 9 shows the changes in weight (W_Loss_) and bloating index (BI) values as a function of the firing temperature for each formulation.

The SCGs present in all the formulations (15 wt%) may be mainly responsible for the changes in W_Loss_. The presence of spent coffee grounds (SCGs), with their inherently high organic content, contributed to a notable increase in weight loss in both firing processes, underscoring their role in affecting the morphological characteristics of the LWAs [8,62]. The variation that occurred in the case of rotary kiln firing was due to the organic fraction present in the clay; as the clay content increased, the weight loss increased. Using static furnace firing, the lowest W_Loss_ value was observed with the lowest clay content and remained fairly stable in the other samples.

Regarding B.I., the aggregate composed entirely of clay had a B.I. value of approximately 24%. Therefore, the B.I. values were all negative, i.e., the aggregates sintered by reducing their size. The presence of pumice inhibited the expansion of the aggregate regardless of the sintering method. However, for the same formulation, the size reduction was greater when fired in a static kiln, indicating a lower degree of expansion during firing, which directly contributed to the final density and porosity of the aggregates [63].

#### 3.1.4. Density, Porosity, Water Absorption, and Compressive Strength

The other properties considered (shown in Table 10) were the following:

- Bulk density (ρ_b_);
- Dry particle density (ρ_Lrd_);
- Loose bulk density (ρ_app_);
- Total porosity (P_tot_);
- Open porosity (P_op_);
- Closed porosity (P_cl_);
- Water absorption in 24 h (WA_24h_);
- Compressive strength (C.S.).

Regarding density, the standard requires that either the dry particle density be less than 2000 kg/m^3^ or the loose bulk density be less than 1200 kg/m^3^. The values given in the table show that all the LWAs fell within the limits and therefore, the obtained aggregates can be classified as lightweight. It can be observed that as the clay content increased, there was a progressive increase in density. The firing method did not particularly affect the obtained values, which are comparable.

The total porosity decreased as the clay content increased, and consequently, when the quantity of pumice decreased. The aggregates fired in a static furnace exhibited a smaller range of variation for this parameter.

The open porosity results did not follow a trend related to the percentages of the raw materials but they were strongly dependent on the amount of water absorbed, a closely related parameter; this was particularly evident with the aggregates fired in a static furnace.

The water absorption results showed a linear trend with increasing pumice content, indicating that the introduction of this scrap contributed to the water retention capacity of the material. This behavior can be attributed to the intrinsically porous structure of pumice.

One parameter that appeared to be related to porosity was the compressive strength (C.S) of the aggregates. A higher clay content typically leads to decreased porosity, which can enhance the compressive strength of aggregates. The experimental formulations incorporating varying ratios of pumice frequently yield superior strength performance due to lower internal porosity and an enhanced density [64]. The compressive strength value of a commercial lightweight aggregate consisting of clay alone is about 2.3 MPa. All the formulations tested showed higher values than that of commercial LWAs, and this was due to the different expansion index values obtained for the tested aggregates (a negative B.I implies fewer cavities in the material skeleton). The different firing methods greatly influenced the strength values, which were higher in the aggregates fired in a static furnace. The prolonged heat exposure in static kilns fosters better sintering of the aggregate materials, decreasing the number of voids and increasing the overall integrity of the materials [65]. This highlights the importance of process optimization in producing lightweight aggregates suitable for agricultural applications.

#### 3.1.5. pH and Electrical Conductivity

A preliminary test to verify the suitability of these porous silicate materials for agronomic use involved monitoring the pH and electrical conductivity of the LWAs obtained through two firing processes. The values for pH must fall in the range of 6.5–7.5 and the E.C. should not exceed 2.0 mS/cm to not damage the soil in which the fertilizers are applied or hinder the growth of plants [22].

**Figure 7:**
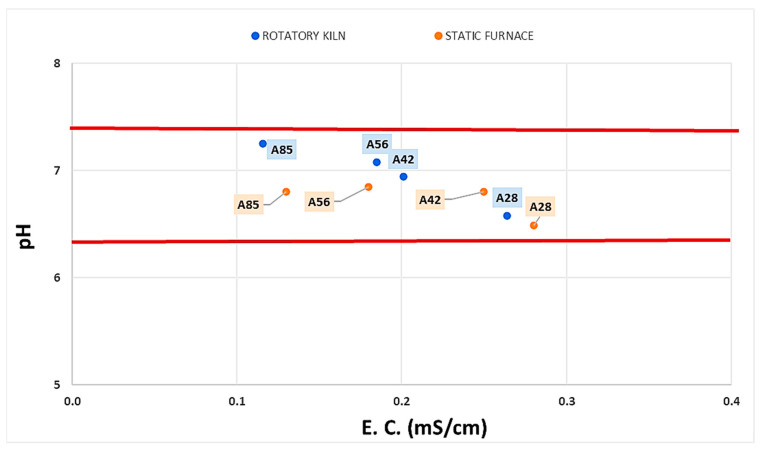
Effect of firing method on pH and electrical conductivity.

The trend in these parameters shown in Figure 7 highlights a non-significant dependence on the firing procedure adopted, but there was a correlation with the formulation. This suggests that the similarity in chemical resistance of the materials is due to the firing procedure.

An increase in the amount of clay at the expense of pumice led to a gradual increase in pH values. The range was between sub-acidic and sub-alkaline (6.5–7.3) but very close to neutral. The pH values found for the analyzed aggregates are compatible with agronomic uses. The firing mode did not affect the pH much, although the samples fired in a static furnace showed slightly lower values.

Electrical conductivity decreased as the clay content increased, but all the values were significantly below the thresholds that could damage crops. The firing mode, again, does not affect the electrical conductivity values.

The assessment of the electrical conductivity and pH levels of the lightweight aggregates indicates favorable properties for agronomic applications. The pH values remained in the neutral range, which is ideal for soil applications, and the low electrical conductivity readings signify that these materials would not adversely affect soil salinity or nutrient availability [66]. These results indicate promise for integrating LWAs into soil systems, facilitating enhanced water retention and aeration conducive to plant growth, aligning with recent studies that emphasize the cultivation of crops in lightweight aggregate-enhanced soils [67].

Lightweight aggregates (LWAs) significantly improve soil aeration and water retention, both of which are essential for plant growth. Thanks to their physical properties, LWAs strike a balance between porosity and weight, thereby improving drainage while retaining moisture. Electrical conductivity tests help to understand how they interact with soil moisture, enabling better design of soil amendments.

LWAs also facilitate smart farming systems by enabling real-time monitoring of soil moisture and nutrients, thereby supporting precision agriculture and reducing resource waste. Additionally, their adjusted electrical properties can stabilize soil, aiding in erosion control and addressing salinity and nutrient issues.

## 4. Conclusions

This study investigated the influence of two different firing methods—using a rotary kiln and static kiln—on the production and performance of lightweight aggregates (LWAs) made from red clay, pumice scraps, and spent coffee grounds. The aim was to explore the feasibility of creating eco-friendly, high-performing materials that could reduce the use of virgin clay while incorporating byproducts and waste and to evaluate the effect of firing method on the LWAs’ properties.

The aggregates obtained using both firing methods demonstrated physical and chemical properties suitable for construction and agronomic applications. In particular, the compressive strength of all the formulations exceeded that of pure clay-based aggregates, although this was accompanied by an increase in the dry particle density. Notably, the A42 formulation, in which 50% of the clay was replaced with pumice, achieved a favorable balance between strength, porosity, and weight. This study demonstrated that using pumice scraps in the composition, instead of clay, could reduce the firing temperature below the conventional range (1100–1300 °C). This further contributes to reducing energy consumption and gas emissions.

Firing in a rotary kiln resulted in aggregates with a more uniform structure, a higher WA% and compressive strength, and a greater dry particle density, largely due to the consistent rotation and improved heat distribution during the sintering process. However, it also produced a negative bloating index, meaning that the aggregates shrank rather than expanded, which led to denser and mechanically stronger materials but with less internal porosity than commercial lightweight aggregates. This method is advantageous for industrial-scale production thanks to its efficiency and ability to deliver consistent quality.

On the other hand, static kiln firing yielded aggregates with even higher compressive strength in some formulations and better resolution of the crystalline phases. However, due to the less uniform heating and longer thermal exposure, this process can lead to more variable porosity and internal structures. Despite this, the mechanical properties of the static kiln-fired aggregates remained excellent, making this method suitable for smaller-scale production or specific applications where uniformity is less critical but mechanical performance is a priority.

Across all the samples, the pH values ranged between 6.5 and 7.3, and the electrical conductivity remained well below agronomic thresholds, indicating the LWAs are safe and effective for use in soil conditioning, green roofs, and hydroponic systems. These properties, combined with their acceptable density and porosity values, confirm compliance with standards for lightweight aggregates and support their suitability for multiple uses.

In addition to the influence of firing conditions, the composition of the aggregates—particularly the proportions of of pumice and spent coffee grounds—was found to significantly impact the final properties. The addition of pumice, a porous and predominantly amorphous material, increased the total and open porosity, thereby enhancing the water absorption capacity. However, it also reduced the bloating index and compressive strength, revealing a trade-off between porosity and structural performance. SCGs, incorporated at a constant 15 wt% in all the formulations, acted as an effective pore-forming agent due to their high organic content, contributing to higher losses on ignition during firing. Despite promoting porosity, the SCGs did not significantly compromise the mechanical strength and helped maintain the pH and electrical conductivity within ranges suitable for agronomic use. Their dual function—as structural modifiers and sources of plant-beneficial nutrients—underscores the value of integrating organic waste into aggregate production. Overall, the combined use of pumice and SCGs enables fine-tuning of LWA properties to meet diverse functional requirements across the construction and agricultural sectors, while also supporting waste valorization and circular economy principles.

In conclusion, lightweight aggregates produced with recycled materials and processed through either rotary or static kiln sintering represent a sustainable and technically reliable alternative to traditional aggregates. The choice of firing method should be guided by the intended application, production scale, and performance requirements: while a rotary kiln is more suitable for industrial-scale, uniform production, a static kiln can yield superior mechanical properties in specific formulations where high strength is needed. These materials contribute to circular economy practices by transforming waste into valuable, multifunctional resources for both construction and agriculture. Future research could further enhance their performance by exploring alternative pore-forming agents or adjusting the ratios of the raw materials.

## Figures and Tables

**Table 1:** Characteristic diameters of pumice rock and spent coffee grounds.

	Pumice Rock	Spent Coffee Grounds
D_10_ (μm)	1.41	30.85
D_50_ (μm)	8.39	192.14
D_90_ (μm)	33.65	434.06

**Table 2:** Chemical analysis (XRF) of raw materials.

Chemical Analysis (wt%)			
Oxide	Red Clay	Pumice	Spent Coffee Grounds
SiO_2_	52.77	56.6	0.01
Al_2_O_3_	17.95	18.6	-
Fe_2_O_3_	7.89	3.9	0.02
MnO	0.19	0.13	-
MgO	3.88	1.17	0.09
CaO	2.57	3.06	0.24
Na_2_O	0.66	1.98	0.06
K_2_O	2.85	8.55	0.89
TiO_2_	0.78	0.54	-
LOI (%)	9.9	4.8	98.11

**Table 3:** Elemental analysis of red clay and spent coffee grounds after calcination.

Elemental Analysis		
%	Red Clay	Spent Coffee Grounds
N	-	2.39
C	0.84	50.28
H	0.75	6.99
S	-	0.08
O	-	38.54

**Table 4:** Heating value of spent coffee grounds.

Sample	Lower Heating Value (kJ/kg)	Higher Heating Value (kJ/kg)
SCG 1	10,837.52	21,698.68
SCG 2	19,775.64	21,636.75
SCG 3	19,715.60	21,576.76
Average value	16,776.25	21,637.40
Standard deviation	5143.18	60.96

**Table 5:** Composition (wt%) of LWAs. The percentage of the optimal water content is to be determined.

Sample	Clay	Pumice Rock	Spent Coffee Grounds	Optimal Water Content
A28	28.3	56.7	15.0	60.7
A42	42.5	42.5	15.0	59.1
A56	56.7	28.3	15.0	45.4
A85	85.0	-	15.0	38.6
A100	100.0	-	-	-

**Table 6:** Working temperatures of rotary kiln.

		Formulations/Temperature (°C)		
A100	A85	A56	A42	A28
1175	1100	1085	1085	1080

**Table 7:** pH of the soils [59].

pH Class	pH
Very acidic	<5.3
Acidic	5.4–5.9
Sub-acidic	6.0–6.7
Neutral	6.8–7.2
Sub-alkaline	7.3–8.1
Alkaline	8.2–8.8
Very alkaline	>8.8

**Table 8:** Electrical conductivity of soils [60].

ECe_1:2.5_ (ms/cm)	Danger of Crop Depression
<0.5	None
0.5–1.0	For sensitive crops
1.1–2.0	For most crops
2.1–4.0	For tolerant crops
>4.0	For all crops

**Table 9:** Comparison of some parameters according to different firing modes.

	Rotatory Kiln				Static Furnace			
	A28	A42	A56	A85	A28	A42	A56	A85
W_Loss_ (%)	20.09 ± 0.12	21.24 ± 0.09	22.02 ± 0.15	23.14 ± 0.05	18.48 ± 0.08	20.66 ± 0.05	19.83 ± 0.05	20.6 ± 0.03
BI (%)	−1.38 ± 0.05	−1.98 ± 0.05	−3.27 ± 0.06	−4.41 ± 0.06	NA	−5 ± 0.09	NA	−5.4 ± 0.08
T Firing (°C)	1100	1080	1085	1080	1000	1000	1000	1000

**Table 10:** Density, porosity, and water absorption of the formulations.

	Rotatory Kiln					Static Furnace			
	A28	A42	A56	A85	A100	A28	A42	A56	A85
ρ_b_ (kg/m^3^)	560 ± 10	620 ± 10	680 ± 10	780 ± 20	460 ± 10	650 ± 10	660 ± 10	720 ± 10	780 ± 10
ρ_Lrd_ (kg/m^3^)	1040 ± 10	1110 ± 10	1210 ± 10	1380 ± 10	860 ± 10	1080 ± 10	1120 ± 10	1180 ± 10	1320 ± 10
ρ_app_ (kg/m^3^)	1380 ± 10	1470 ± 10	1650 ± 10	1830 ± 10	940 ± 10	1550 ± 10	1530 ± 10	1520 ± 10	1660 ± 10
P_TOT_ (%)	60.13 ± 0.02	57.18 ± 0.02	53.48 ± 0.02	46.94 ± 0.03	58.60 ± 0.01	58.34 ± 0.01	56.99 ± 0.01	54.82 ± 0.01	49.20 ± 0.02
P_op_ (%)	24.87 ± 0.01	24.24 ± 0.01	26.72 ± 0.01	24.74 ± 0.01	3.80 ± 0.01	30.22 ± 0.01	27.05 ± 0.01	22.71 ± 0.01	20.43 ± 0.01
P_cl_ (%)	35.26 ± 0.01	32.94 ± 0.01	26.77 ± 0.01	22.20 ± 0.01	54.80 ± 0.01	28.12 ± 0.01	29.94 ± 0.01	32.11 ± 0.01	28.77 ± 0.01
WA_24h_ (%)	23.91 ± 0.10	21.71 ± 0.08	22.03 ± 0.08	17.88 ± 0.09	4.40 ± 0.10	27.82 ± 0.11	24.12 ± 0.10	19.28 ± 0.09	15.42 ± 0.10
C.S (MPa)	3.30 ± 1.47	3.80 ± 1.08	4.40 ± 0.89	6.90 ± 0.90	2.30 ± 0.63	4.80 ± 2.49	5.90 ± 2.31	8.60 ± 1.97	10.80 ± 1.67

## Data Availability

The original contributions presented in this study are included in the article. Further inquiries can be directed to the corresponding authors.
